# INCENTIVES + SOCIAL MEDIA RECRUITMENT + MINIMAL SUBJECT INTERACTION = POTENTIAL FOR FRAUD!

**DOI:** 10.1093/geroni/igad104.1178

**Published:** 2023-12-21

**Authors:** Joan Carpenter, Casey Jackson, Liza Behrens

**Affiliations:** University of Maryland School of Nursing, Baltimore, Maryland, United States; University of Maryland, Baltimore, Maryland, United States; Pennsylvania State University, State College, Pennsylvania, United States

## Abstract

Social networking technologies create opportunities to recruit a more diverse and representative research sample. However, social media recruitment strategies with low researcher interaction for screening brings threats to data integrity, especially when incentives are involved. During a recent study designed to ascertain direct care nursing home staffs’ perceptions of risk during the COVID-19 pandemic, we experienced fraudulent research participants. We aimed to recruit, enroll, and collect survey and semi-structured interview data from N=30 participants between June 2021 through March 2022. Participants were compensated $25. Initial recruitment efforts targeted Facebook listservs for licensed and unlicensed nurses, social workers, and activity directors. Research staff provided participants who screened eligible for the study via email a link to initiate consent and complete the survey electronically. Four months into study recruitment, study staff discovered during an interview that one participant was unable to answer questions in the correct context as a direct care worker and responses were nonsensical. Additional quality control measures confirmed that “bots” had completed surveys (N=8). The fraudulent data were removed from the study database and corrective actions implemented into screening to rule out ineligible participants/bots including screening for repetitive language and grammatical errors in email conversations, verifying eligibility through phone communications, and adding a Captcha verification prior to electronic survey consent. Our experiences highlight the importance of creating study protocols that include social media recruitment with safeguards to fraudulent participants. This is especially important for investigators attempting to recruit hard to reach populations and participants who wish to remain anonymous.

